# Diet-Derived Phytochemicals Targeting Colon Cancer Stem Cells and Microbiota in Colorectal Cancer

**DOI:** 10.3390/ijms21113976

**Published:** 2020-06-01

**Authors:** Kumar Ganesan, Muthukumaran Jayachandran, Baojun Xu

**Affiliations:** 1 Food Science and Technology Programme, Beijing Normal University-Hong Kong Baptist University United International College, Zhuhai 519087, China; kumarg@hku.hk (K.G.); jmkbio1@gmail.com (M.J.); 2 Laboratory and Clinical Research Institute for Pain, Department of Anesthesiology, The University of Hong Kong, Hong Kong SAR, China

**Keywords:** phytochemicals, gut microbiota, colon cancer stem cells, CRC therapy

## Abstract

Colorectal cancer (CRC) is a fatal disease caused by the uncontrolled propagation and endurance of atypical colon cells. A person’s lifestyle and eating pattern have significant impacts on the CRC in a positive and/or negative way. Diet-derived phytochemicals modulate the microbiome as well as targeting colon cancer stem cells (CSCs) that are found to offer significant protective effects against CRC, which were organized in an appropriate spot on the paper. All information on dietary phytochemicals, gut microbiome, CSCs, and their influence on CRC were accessed from the various databases and electronic search engines. The effectiveness of CRC can be reduced using various dietary phytochemicals or modulating microbiome that reduces or inverses the progression of a tumor as well as CSCs, which could be a promising and efficient way to reduce the burden of CRC. Phytochemicals with modulation of gut microbiome continue to be auspicious investigations in CRC through noticeable anti-tumorigenic effects and goals to CSCs, which provides new openings for cancer inhibition and treatment.

## 1. Introduction

Colorectal cancer (CRC) is one of the most fatal diseases and foremost causes of death globally, representing the third most common malignancy. The American Cancer Society estimated that the rough sum of CRC incidences in the United States in 2018 alone was 97,220 (colon cancer), and 43,030 (rectal cancer), which had a great influence on curative care, which exceeded $17 billion in the medical care system [1]. The CRC develops (70%) via a serious transformation of specific morphological traits, denoted as adenoma to a carcinoma sequence [2]. About 30% of CRC cases are caused due to hereditary disorder, often connected with familial adenomatous polyposis and/or hereditary non-polyposis [3]. Chronic inflammatory bowel diseases (IBD) or family history of CRC are the primary causes of CRC [4]. In economically developed countries, the mortality connected to CRC is greater than the economically developing nations, and it affects over a million people annually [5]. Several epidemiological studies have also shown different risk factors to CRC including age, family history, IBD, obesity, smoking, lack of exercise, alcohol consumption, and diet [6]. Unfortunately, the present treatments are inadequate, owing to its effective treatment, and besides, have various side effects, chemo-resistance, and recurrence of the illness.

The growing oncogenic study provides awareness about the malignancies in humans that could have a history of stem cell diseases. Rendering to the cancer stem cell (CSC) study, CRC originates from a minor portion of tumor cells in the colon that demonstrate self-renewal, pluripotency, and can recruit and sustain tumor development [7]. The cancer-developing cells or CSC were initially recognized in blood cancer, which is copious in most of the hard tumors, especially in CRC. The smaller fraction of CSC can develop the spread of tumorous tissues, in analog to target tissues that produce effective histological units, and organs. CSCs are generally tumor-initiating, self-renewal, long-lasting cells that divide asymmetrically and harvest aggressively thriving cancer progenitor cells. These cells are resistant to cytotoxic conditions, divide into the manifold, and create endless copies, characterizing clinically relevant CRC development [8]. Nowadays, the prevalence of CRC is increasing even in historically low-risk nations, including Korea, Japan, China, and Eastern Europe. A high-frequency rate of CRC has been reported in these topographical areas, which is due to the outcomes of western diets, microbiota alterations in the gut, and cancer-causing dietary components [2,9]. Being overweight and obesity are also recognized risk factors of CRC. High consumption of red meat and reduced intake of fruits and vegetables are additional key factors to the increase f the menace of CRC [10].

To alleviate the effects of CRC and understanding the colon CSCs proliferation, there is an urgent requirement to develop an innovative and safer drug for treating CRC and preventing CSCs growth. Recently, diet-derived phytochemicals or bioactive compounds have the potential to reduce the effects of CRC that upsurge many interests among researchers [7,11]. Recently, the impact of phytochemicals in decreasing the risk of CRC and the connection with CSCs are well-documented in the literature [12,13,14]. The actions of bioactive compounds are varied depending on distinct chemicals by targeting diverse pathways and beneficial to human health. Various preclinical investigations have been examined related to anti-cancer activities of phytocompounds in CRC models. The results suggest several novel compounds such as apigenin, betanin, *α*, and *β*-carotene, diallyl sulfide, ethyl gallate, gallic acid, resveratrol, quercetin, luteolin, silymarin [15,16]. These compounds are harmless and can be employed in synergistic treatment to decrease cancer cell growth via chemotherapeutic mediators [15].

The microbiome is comprised of the main inhabitants in the human gut, comprising of 100 trillion microbes with diverse actions that maintain the integrity of a healthy colon [17]. Undigested dietary residues in the colonic lumen are the prime energy sources for the gut microbiota, which digest those dietary residues, resulting in the formation of several active metabolites with favorable functions. Imbalance of gut microbiota or dysbiosis can lead to several pathologies, including infectious diseases, gastrointestinal cancers, inflammatory bowel disease, and even obesity and diabetes. Dysbiosis may cause chronic inflammation, recognized as one of the prime causes of CRC. Earlier, our publications have also summarized the functions of gut microbiota, particularly, short-chain fatty acid synthesis with their benefits to the hosts in regulating various diseases such as diabetes, cardiovascular diseases, and cancer [18,19,20]. Dietary interventions or the consumption of phytochemicals is the beneficial component, which has been proved as effective in treating CRC [21,22,23,24,25,26,27,28]. Taking this into account, we aimed to review in-depth analysis of various diet-derived phytochemicals mediating the gut microbiome and its role in CRC prevention and treatment. In addition, we intend to review the dietary phytochemical interventions targeting colon CSCs on CRC prevention.

## 2. Diet-Derived Phytochemicals Modulate the Gut Microbiome

Earlier studies suggested the gut microbiota (*Bacteroides fragilis*, *Escherichia coli* strain NC101, *Desulfovibrio, Helicobacter hepaticus*, *Clostridium ramosum*, Fusobacterium, Campylobacter, *Prevotella*, etc.) in humans play a significant role and alter the immune function through pro-carcinogenic markers resulting in the etiology of CRC [20]. Altering the immune system in the gut normally enhances tumor microhabitats, and inflammation, ensuing the CRC development [19]. In recent research has also recommended genetically reformed colon bacteria, which are beneficial and are currently employed in experimental cases that outcomes are promising [29]. Furthermore, they can be greatly beneficial to the host as probiotics that inhibit CRC through alterations of microbiota and colon environment.

The consumption of natural products produces essential bioeffects in the body through multifaceted relations with gut microbiota [30,31]. Natural phytochemicals normally have fiber-rich glycosides that exist as complex molecules with the properties of lower bioavailability and lesser solubility [32]. The nature of the phytochemicals could be altered during microbial fermentation in the colon, ensuring high quantities of various byproducts with greater pharmacological activity [33]. Numerous metabolites that derived from gut microbiota may further be subject to various enzymatic cleavage by methylation, glucuronidation, glycination, or sulfation in the hepatocytes, which are then trafficked into the tissues and finally excreted into the gut [32,34]. Gut microbiota converts glucuronides to aglycones by *β*-glucuronidases, which can be immediately reabsorbed in the colon. Thus, the synthesis of microbial *β*-glucuronidase and its enterohepatic passage have possible steps to extend the holding period of phytochemicals in the host [32,34]. Rising data suggested the dietary phytometabolites derived from gut microbiota, which are capable of enhancing the bioavailability, antioxidant properties, detoxification of xenobiotics, and prebiotics function [34,35]. Furthermore, these compounds can eliminate gut pathogenic organisms, reduce oxidative DNA damage and pro-inflammatory mediators, and thus regulate normal cell division and apoptosis [36,37]. The effects of phytochemicals on gut microbiota and their anti-inflammatory effects are presented in Table 1.

### 2.1. Polyphenols

Polyphenols are one of the prime classes of chemicals in plants, extensively studied for their health-promoting properties [38,39,40]. Human diets contain varieties of polyphenols and have significant protective activities against various cancer types. Scavenging of free radicals and reducing oxidative stress are the key mechanisms by which a polyphenol can achieve [38]. Several studies confirmed the actions of polyphenols on CRC inhibition, which often interconnected with the relationship of gut microbiota [41,42,43]. For instance, an animal study was conducted related to cranberry polyphenols on *Akkermansia* (mucin-degrading bacterium), which protected the host from obesity, diabetes, and gut inflammation. In this study, the mice were administered with high fat and sugar diet and cranberry extract (CE) (200 mg/kg/day) for eight weeks, and the various gut microbiota were analyzed by the methods of 16S rRNA and 454 pyrosequencing. The outcomes of the study revealed the administration of CE reduced body weight, visceral fat obesity, triglyceride accumulation, and inflammation, and elevated antioxidant properties and insulin sensitivity. Furthermore, the metagenomics study of CE treatment exhibited an increased percentage of *Akkermansia* [42].

The anti-carcinogenic properties of the gut microbiota are generally attributed based on the two properties, (a) either by improving the host’s immune system or (b) by generating the metabolites, which can interfere with the pathways involving CRC formation. A study demonstrated that the presence of amines, bile acids, and high consumption of meat can reduce some bacterial growth such as *Clostridium,* which inhibits the development of CRC [43]. By using the alimentary metabolites, gut microbiota produces biologically active short-chain fatty acids. The *Rosburia faecis* and *Eubacterium rectale* group of bacteria can normally produce the butyrate, which involves reducing cell apoptosis and diversity [41]. A study showed the polyphenol metabolites modulated microbiota that directly restricted the growth/proliferation of CRC [44]. Another study has also related intestinal metabolites, quercetin, chlorogenic, and caffeic acids to interfering in cyclooxygenase-2 expression resulting in the prevention of DNA damage in the colon [45]. The polyphenols-mediated gut microbiota changes are a potential technique for inhibiting colon cancer, although insufficient trials have been piloted, in which, wine [46], blueberry [47], and cocoa [48] displayed a bifidogenic outcome.

### 2.2. Flavonoids

Flavonoids are mainly present in fruits, vegetables, seeds, and various beverages such as tea, coffee, and red wine. Several medicinal herbs are amongst the richest sources of flavonoids. They are grouped into the following sub-classes-flavonols (quercetin, rutin), flavanols (catechin, epicatechin, and epigallocatechin), flavones (luteolin, apigenin), anthocyanidins (malvidin, cyanidin, and delphinidin), isoflavones (daidzein, genistein, glycetin, and formanantine), and flavanones (naringenin, hesperetin) [70,71]. A hypothesis stated that the presence of beneficial phytochemicals in diets attributes an anticancer property to the respective food. The flavonoids present in the food prevent CRC development by exerting various mechanisms: alleviating DNA damage, reducing the effects of gene mutation, regulation of phase I, and phase II enzymes via modulation in cell signaling pathways, suppressing oncogene expression, and regulating inflammatory responses [72,73,74,75,76]. In a recent clinical trial, a flavonoid mixture of 20 mg apigenin along with 20 mg epigallocatechin gallate was given to CRC patients daily for long-term interventions that showed the reduction of CRC relapse [77]. The greater quantities of polymeric flavonoids and the non-absorbed flavonoids passed into the colon region where they underwent breakdown and gut microbiota facilitate converting these flavonoids into simple phenolic acids [78].

The digestion of flavonoids is often mediated by gut microbiota, which is a similar pattern to other phytochemicals. Gut microbiota facilitates converting a large group of flavonoids into simple active metabolites (aromatic catabolites and small phenolic acids) by oxidation and demethylation [14,79]. These active products augment physiological activity and perform various roles in the regulation of the host’s immune system. One best instance for the gut microbiota-mediated metabolite is daidzein-isoflavones, which serves various benefits to the host. Daidzein is found in numerous plants and predominantly occurs in soybeans; daidzein is transformed by bacterial flora into the most active compound equol. In vitro and clinical trials showed that equol is more bioactive than daidzein (food precursor), and the biological effect is significantly improved in patients who produced equol after isoflavone consumption [80]. This result strongly suggested that gut microbiota aid a pivotal function in regulating the biological effects of ingested phytochemicals.

We recognize that the impacts of the gut bacterium on the flavonoids and the effects of flavonoids on the gut microbiota are bidirectional. Flavonoids can change the organization and roles of gut microbiota, and similarly, gut microbiota can enhance the flavonoid breakdown. A case pilot study with 178 elderly people showed the habitual diet, which contributed to bacterial alterations resulted in the improvement of frailty and inflammation [81]. Another fascinating study revealed that 15 women with a two-month dietary intervention connected to alterations of gut microbiota including, Gammaproteobacteria and Erysipelotrichi [82]. A study on the impacts of grape extract (GE) on experimental animals showed the reduction of the Firmicutes-to-Bacteroidetes ratio and an increasing of *Akkermansia muciniphila*. Supplementation of GE along with gut microbiota significantly reduced inflammatory response and improved insulin sensitivity. These findings offered noteworthy support in favor of colonic bacteria and their substantial role in facilitating the flavonoids on health impacts, which reduced inflammatory response as well as improved the metabolic function. Another interesting clinical study demonstrated that the feeding stable isotope-labeled anthocyanins were ingested by gut microbiota, which yielded high quantities of diverse active metabolites [17,83]. These colonic bioactive phytometabolites exert greater anti-inflammatory functions and maintain vascular integrity when compared to the normal colonic metabolites [84]. This statement complements the belief of the effect of increased activities of phytochemicals on host health, which are the utmost prospective study related to gut microbiota.

## 3. Colon CSCs and their Tumorigenic Effects

Over the last decade, the development CSC model has progressively recognized as an account for cancer propagation and recurrent. The CSC model was initially established for hematological malignancy and in recent years, many investigators validated it for other solid tumors, including colon CSC [85,86,87]. This model proposed a salient feature of the CSCs: minor populace of colonic cells, greater strength, capacity to recruit distinct metastases, capable of self-renewal, becoming metastatic heterogeneous tumors, and more resistant to various therapies [85]. During an asymmetric division, these multipotent cells generate populace cells without any control measures contributing to tumorigenesis. Loss of cell replicative control usually leads to an increased count of cells like embryonic stem cells that lead to tumor growth [87]. These stem cells and their offspring can harbor an astonishing number of inconsistent cells based on the DNA mutations, which may contribute heterogeneous tumors and carcinogenesis [88].

Colon cancer primarily increases through abnormal directions of the Wnt/*β*-catenin pathway, either activating mutations in *β*-catenin or disabling mutations in the *β*-catenin regulator, adenomatous polyposis coli (APC). This mechanism provides irregular deposition and stimulation of a *β*-catenin/transcription factor T-cell factor 4 (Tcf4) in the nucleus, which targets c-MYC resulting in the prevention of p21CIP1/WAF1 expression [86]. Notch and Hedgehog (Hh) pathways have also presented to be intricate in the maintenance of the self-renewal in either a normal stem cell or colon CSC [87]. The Wnt pathway contributes to CSC proliferation through the prevention of GSK-3β, phosphorylation of *β*-catenin, endorses its translocation to the nucleus, and activates Tcf4 [89]. Animal trials have also confirmed that activated *β*-catenin spread to the cell and become malignant [90].

Various researches confirmed that the intestinal markers contributed to characterizing and distinguishing normal colon stem cells from colon CSC [91,92]. Normal colon stem cells are identified by various markers such as Msi-1, Hes1, integrins α2, and β1 subunits, EphB receptors, Bmi-1, Lgr5, and Aldh1, whereas colon CSC is recognized by CD44, CDD133, CD166, CD34, CD24, ESA, LGR5, CD29, nuclear *β*–catenin, EpCAM, CD49f and Aldh1 [91,92,93]. Colon CSC markers are often used as prognostic indicators that help eliminate colon CSCs. The list of the disease model, markers, and the mechanism associated with the findings presented in Table 2. Several genes and their multiple signaling pathways have been identified in normal and colon CSC. Inconsistency of these cellular signaling triggers anomalous transformation, tumorigenesis, resulting in cancer. The major pathways, Notch, Hh, and Wnt/*β*-catenin participate in the maintenance of the self- renewal of both SCs and CSCs, where Hh is a glycoprotein, involved in the pro-survival pathways; Notch and Wnt/*β*-catenin involve in the self-renewal [89].

## 4. Effect of Diet-Derived Phytochemicals on the CSCs

**Figure 1:**
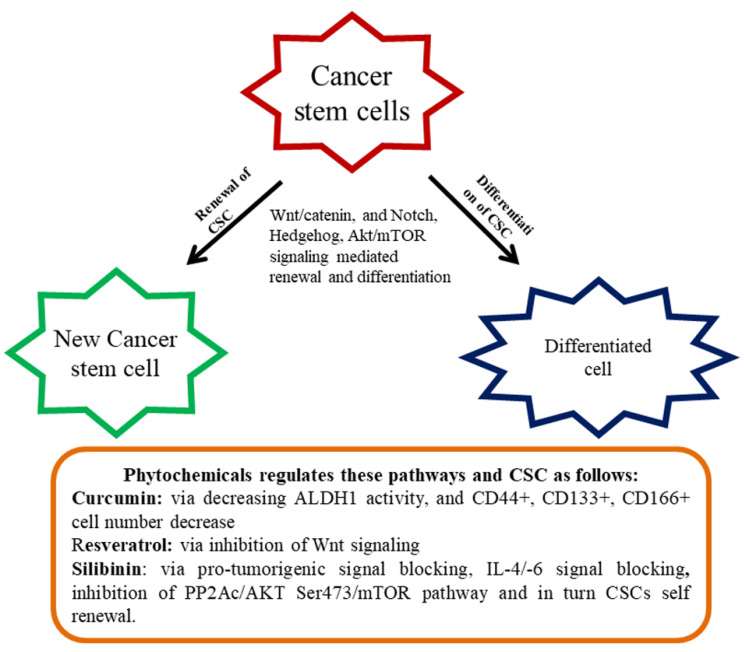
Renewal and differentiation of cancer stem cells (CSC). Diet-derived phytochemicals generally attenuate various signaling mediated renewal and differentiation and thereby regulate CSC proliferation.

Signal transduction pathways, namely, Hh, Wnt/*β*-catenin, and Notch, contribute to a variety of usual stem cells and provide striking strategies to CSC [101,102]. Irregular cascade signaling of Wnt/*β*-catenin causes the majority of malignancy in most individuals [103]. Preclinical investigations have been undertaken to find small molecules, which are capable of distracting the pathway of Wnt/*β*-catenin [104,105]. Based on the findings, monoclonal antibodies and siRNA are promising blockers against the Wnt1/2 pathway [104,105]. However, targeting Wnt1/2 is still a primitive stage and no beneficial mediators have yet been permitted for patient practice until today [106]. Numerous bioactive chemicals have been studied in inhibiting the above-stated pathways. For example, Corn lily-derived cyclopamine that targeted hedgehog signaling [107]. Epigallocatechin gallate (EGCG) inhibited Wnt/*β*-catenin signaling and was found to influence CSC self-renewal and invasive abilities [108,109]. Retinoic acid is an active molecule derived from vitamin A, can downregulate the Notch signaling, and differentiate CSCs or reduce their development [110]. The Akt/mTOR signaling pathway is one of the significant pathways intricate in the CSC. This CSC existence and invasion of the stimulation of Akt/mTOR is very decisive. Declining motility and apoptosis commencement of CSC occurs repetitively, owing to Akt deterrence [35] (Figure 1).

The anti-cancer effect of polyphenols is normally achieved by the inhibition of tumor cell proliferation, and stimulation of caspase-3-dependent apoptosis via the Akt/mTOR pathway [111]. An assortment of the investigation suggested that polyphenols and flavonoids can affect various CSCs and inhibit proliferation and thus the outcomes exhibited phytochemicals are promising anti-cancer agents targeting CSCs [112]. There are several colon CSCs markers with varying functions comprising a cluster of differentiation 44 (CD44, a receptor of hyaluronic acid), CD133 (unidentified), CD166 (fixative substances), and aldehyde dehydrogenase-1 (Aldh-1 an enzyme). In tumors, CD133 is recognized as a colon cancer-originating cell. The markers of CD166 along with CD4435 or CD24/CD29 identified the populace of colorectal CSC. Aldh1 is also accepted as a novel indicator of CSCs in humans. Curcumin contributed to the control of colon CSCs and standardized the several markers of CRC stem cells. It reduced Aldh1, CD44+, CD133+, CD166+ cell numbers, and enhanced apoptosis in tumors [113]. In another study, curcumin-enhanced G2/M phase arrested and downregulated *β*-catenin expression [114].

An interesting study on the *Sasa quelpaertensis* extract (SQE) showed the induction of CSC variation and inhibited Wnt signaling. SQE contains high quantities of polyphenol, including *p*-coumaric acid and tricin that inhibited the renewal and differentiation of CSC [115]. In this study associated with colon, HCT116, and HT29 CSCs were labeled with respective markers (CD133+ and CD44+) and introduced into the nude mice to develop the CRC. The nude mice were administered with the SQE extract (300 mg/kg b.w) that reduced signaling of CSC marker expression and Wnt/*β*-catenin, as well as the hypoxia-inducible factor-1α [115]. Resveratrol, a renowned phytochemical present in several dietary sources inhibited the effect on colon CSCs through the hindrance of Wnt signaling [116]. Ellagic acid is an active principle of walnut displayed to inhibit CRC by regulating the colon CSCs [117]. Silibinin is another imperative phytochemical revealed to regulate colon CSCs via blocking of pro-tumorigenic signaling, including, IL-4/IL-6 [118]. By overwhelming the PP2Ac/AKT Ser473/ mTOR pathway, silibinin impeded colon CSCs self-renewal [92]. In an interesting study connected to the colon CSC, cinnamic acid found to reduce the CSC markers connected with HT-29 colon cancer cells [119].

## 5. The Anti-Tumorigenic Potential of Phytochemicals through Various Molecular Goals in Colon CSC

**Figure 2:**
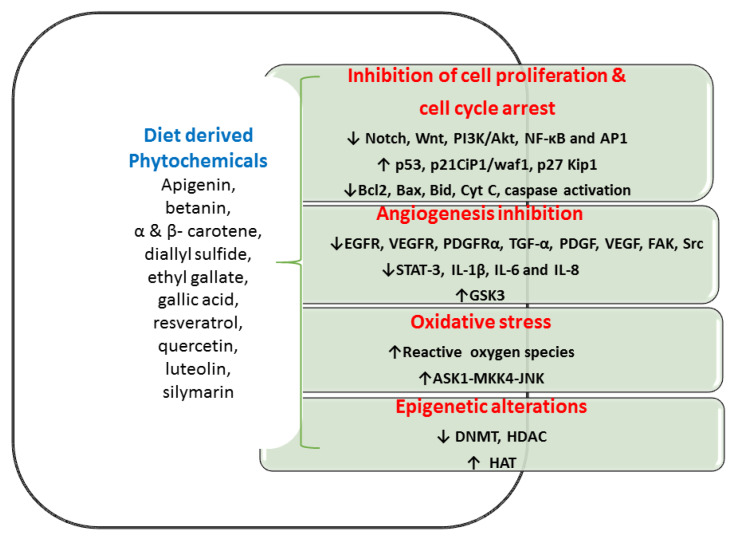
Various in vitro and in vivo studies showed the phytochemicals inhibit cell propagation, differentiation, angiogenesis, and anti-apoptotic activities in the colon. Abbreviation: Akt- serine/threonine-specific protein kinase; AP1—Activator protein 1; ASK1—apoptosis signal-regulating kinase 1; Bax—bcl-2-like protein; Bcl 2-B-cell lymphoma 2; Bid—BH3 Interacting Domain Death Agonist; CIP1/waf1—cyclin-dependent kinase inhibitor 1; Cyt C—cytochrome C; DNMT—DNA methyltransferase; EGFR—epidermal growth factor receptor; FAK—Focal adhesion kinase; GSK3- glycogen synthase kinase-3; HAT—histone acetyltransferases; HDAC—histone deacetylase; IL- interleukin; JNK—c-Jun N-terminal kinases; Kip1—kinesin-like protein1; MKK4—mitogen-activated protein kinase kinase 4; NF-κB—nuclear factor kappa-B; PDGF—platelet-derived growth factor; PDGFRα—platelet-derived growth factor receptor A; PI3K—Phosphoinositide 3-kinases; SrC—protooncogene c; STAT3—signal transducer and activator of transcription 3; TGFα—Transforming Growth Factor-alpha; VEGF—vascular endothelial growth factor; VEGFR—vascular endothelial growth factor receptor.

Globally, diet-derived phytochemicals lead to reduced CRC incidences. For incidence, Mediterranean people generally have a low prevalence of CRC, because of the high consumption of olive oil and tomato [120]. Both olive oil and tomato have phytochemical-rich dietary materials that can reduce CRC in Mediterranean individuals [120]. Various in vitro and in vivo studies showed that phytochemicals inhibit cell propagation, differentiation, angiogenesis, and anti-apoptotic activities in the colon (Figure 2 and Table 3). These diet-derived phytochemicals offered a significant success rate in numerous medical trials of CRC individuals [121,122,123]. A beneficial efficacy of diet-derived phytochemicals in the CRC management especially targeting of CSCs increases greater interests among researchers [124,125]. Antitumor effects of diet-derived phytochemicals are presented via four molecular targets as below.

### 5.1. Inhibition of Cell Multiplication and Cell Cycle Progression

Colon CSCs have the ability of proliferation and metastatic effect with atypical maintenance of numerous signaling pathways, accountable for malignancy. Diet-derived phytocompounds that are connected to multiple signalings, such as PI3K/Akt, Hh, Wnt, and Notch could be beneficial healing approaches in managing CSCs induced malignancy. The unusual stimulus of NF-κB signaling normally accelerates malignant cell proliferation that averts apoptosis [130]. Phytocompounds contribute to the initiation of this apoptosis, prevent cell division with cell cycle growth, and hence phytocompounds are a great attractive drug candidature for tumor therapy. Various cancer models connected with phytochemicals that have established with the upregulation of proapoptotic proteins (Bax, and Cyt C), triggers caspase cascade, and cleavage of poly (ADP-Ribose) polymerase and thus regulates cancer development [105,131]. Diet-derived phytochemicals such as curcumin, EGCG, and lycopene demonstrated an ability to increase apoptosis via induction of p53-dependent Bax, upregulating p21waf1/Cip1, and p27Kip1 CDK inhibitors and thus repressed the normal cell cycle [132,133].

Likewise, isothiocyanates exhibited a reduction in the incidence of CRC through elevated apoptosis, cessation of the cell cycle, and self-renewal of CSCs [15]. Curcumin, gingerol, EGCG, and resveratrol inhibited the signaling of Notch, Wnt signaling, *β*-catenin/TCF transcription as well as targets to avert CSC self-renewal [134,135]. Sulforaphane is generally acquired from broccoli, which is effective in preventing colon CSCs proliferation through modulation of multiple signaling pathways, comprising PI3K-Akt, NF-κB, Hh, Wnt/*β*-catenin [136,137].

### 5.2. Inhibition of Angiogenesis Mechanism

Angiogenesis supports CRC initiation, development, and metastasis and its suppression provides a fascinating strategy for the treatment of CRC. Diet-derived phytochemicals reduce angiogenesis through several pathways. Curcumin gingerol, and EGCG inhibited Wnt signaling with various receptors of the epidermal growth factor (EGFR), vascular endothelial growth factors (VEGFR-1, VEGFR-2, and VEGFR-3) and downregulated IL-1β, IL-6, and IL-8 and thus these compounds inhibited chemoresistance, angiogenesis, and invasion [138]. Experiments validated that dose-dependent manners of curcumin prevented interleukin from the gut and inhibited angiogenesis and CSCs stimulation [138]. EGCG impeded angiogenesis and growth of the tumors through the activation of receptors of EGFR and platelet-derived growth factor receptor-α (PDGFRα) [139]. Studies established that capsaicin inhibited CRC-provoked angiogenesis through the reduction of the STAT-3 facilitated downstream mechanism [140]. Isoflavones also suppressed Wnt signaling by augmenting glycogen synthase kinase expression, fixes with *β*-catenin resulting in elevated phosphorylation, and successively decreased CRC development [141].

### 5.3. Oxidative Stress and Anti-Tumorigenic Effect

Investigators established that CSCs in many tumor cells contain a negligible concentration of reactive oxygen species (ROS) and these quantities are dynamic for preserving normal stem cell functions [142]. These ROS conservations in normal cells as well as CSCs are greatly important. The beneficial outcome of elevated ROS eradicates CSCs, which can be one of the vital goals for CRC treatment. Hence, the increased ROS plays as “double-edged sword”, which is not only an illness maker but also as a missile in tumor treatment. Curcumin has contradictory roles in hunting and creating ROS, and however, the consumption of dietary curcumin possesses a potential anticancer activity. Curcumin-induced ROS generation and their oxidative stress that largely induced cell apoptosis in HT29 cell lines through the activation of signaling cascade ASK1-MKK4-JNK [143]. Studies revealed the twin function of lycopene as a ROS scavenger and creator based on its dose-dependent manner. Ribeiro et al. [144] established oxidative stress in the HT29 cell line, resulting in functional DNA impairment, which was greatly secured by lycopene (1-3 μM); however, DNA damage is amplified while lycopene treatment in higher concentrations (4-10 μM). Capsaicin-stimulated apoptosis in human CRC cell lines, which is connected with an upsurge production of ROS and disruption of membrane potential in mitochondria [145].

### 5.4. Epigenetic Alterations

There are three enzymes viz., DNA methyltransferases (DNMTs), histone acetyltransferases (HATs), and histone deacetylases (HDACs) play an energetic function in chromatin organization and direction of transcription. HAT activity is connected to dynamic chromatin in transcription, while, DNMTs and HDACs induce silencing of the gene. The disparity of DNA methylation and histone acetylation/deacetylation often contributes to cancer. Multiple signaling pathways in CRC comprise Wnt/β-catenin, Hh, Notch, and TGF-*β*/BMP provide self-renewal, and variation in stem cells that are regularly modulated by epigenetic mechanisms. The mechanism of HDAC inhibition is an extensive platform of anti-tumorigenic effects comprising cell cycle arrest, apoptosis, and cell differentiation that have fascinated new consideration as possible anticancer candidates. Various researchers have recommended that curcumin, phenyl isothiocyanate, EGCG have anti-tumorigenic properties that are possibly mediated through an epigenetic mechanism by DNMTs and HATs inhibition [146,147].

## 6. Effect of the Gut Microbiome on Colon CSCs and CRC

**Figure 3:**
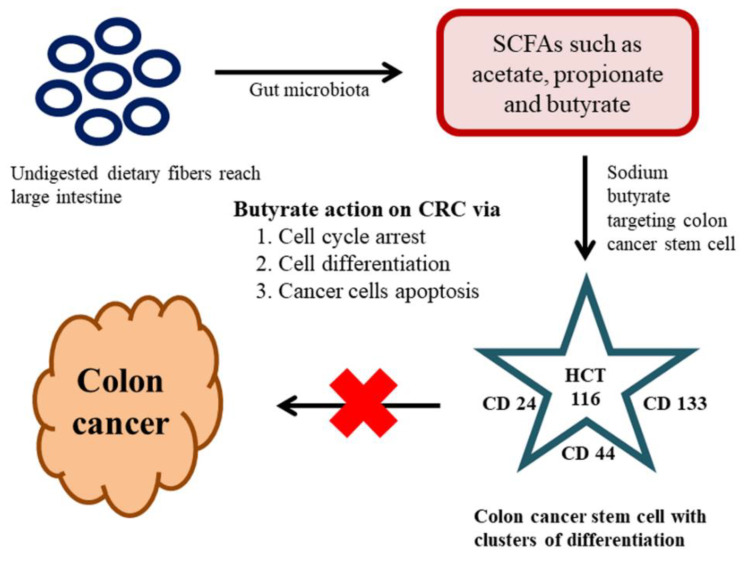
Effect of the gut microbiome on colon cancer stemm cells (CSCs) and colorectal cancer (CRC).

The key factors contributing to CRC are colon CSCs and diet, which is a renowned and significant environmental factor connected to CRC. The metabolites from gut microbiota have the potential to be either tumorigenic or anti-tumorigenic agents. Intestinal microbiota produces short-chain fatty acids (SCFAs) from the dietary fibers through fermentation in the host. SCFAs are aliphatic carbon-based acids, in which the most abundant SCFAs are acetate (C2), propionate (C3), and butyrate (C4) [148]. These SCFAs are shown to exert numerous beneficial effects on the host’s energy metabolism. The dietary fibers reach into the large intestine without undergoing any course of digestion; the reason is owing to the absence of dietary digestive enzymes in the upper intestinal tract. The gut microbiota present in the large intestine is accountable for the breakdown of these dietary fibers into active metabolites [149]. One noteworthy beneficial effect of SCFAs with over the host immune system is butyrate. Butyrate is a metabolic product of dietary fiber and resistant starch by the bacterial action (*Faecalibacterium prausnitzii* and *Eubacterium rectale*) in the colonic lumen. The literature claimed that the butyrate can induce G1 phase-cell cycle arrest, cell differentiation, and apoptosis in CRC [150]. As we stated earlier only a small populace of cells is accountable for the initial generation of malignancy cells and referred to as CSCs. The method of traditional malignancy therapy is proven ineffective against these CSCs. The CSC holds a specific cluster of differentiation (CD) markers on their surface, hence targeting those CD markers may be the best way to target the CSCs [151]. In a recent study on the effects of butyrate on the colon CSCs, sodium butyrate (NaB) employed in CRC stem cells (human) type HCT116. The investigation was carried out by analyzing the expression profiles of a definite marker for CRC stem cells, including CD24, CD133, and CD44. These results revealed that the SCFA-NaB had variable impacts on HCT116 stem cells (CD24, CD44, and CD133). The results still varied bestowing on the concentration of NaB and incubation time. Overall, this study offers some interesting information on NaB and whether it is possible to develop it as a novel therapeutic drug targeting cancer stem cells [152] (Figure 3).

**Figure 4:**
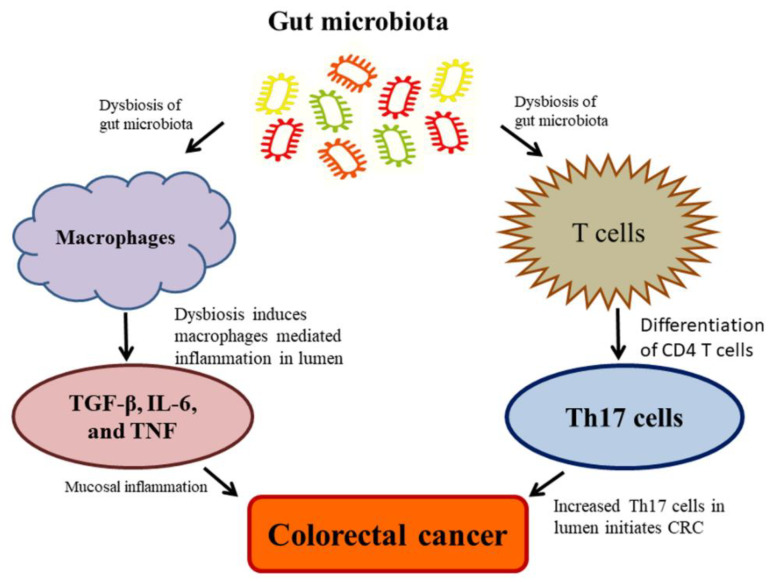
Dysbiosis of gut microbiota causes a high risk of colorectal cancer (CRC).

Currently, the studies on the complexity of microbiota associated with CRC revealed that microbiota unconditionally influences CRC at high risk due to the range and complexity of the gut microbiota. Despite the contemporary debate, regarding whether alterations in the microbiota give rise to colon carcinogenesis, in which, some noteworthy explanations have been made to recommend a causative function of the gut microbiota in the CRC. The rodent model investigations employed as natural, chemically-induced, or genetically predisposed CRC, which revealed the enhancing tumorigenic properties of microbiota and their effects on the development of CRC [97,100]. These tumorigenicity effects are attributed to inflammation, which plays as a cancer inducer in animals. Dysbiosis has also detected during the exposure of subsequent radiation in animals, which represented the vulnerability of the microbiota; additionally, dysbiosis may play as a pilot and facilitate the formation of CRC [153] (Figure 4). Eubiosis is referred to as the balance microbiome status maintaining healthy human body conditions [154]. Upon the impaired eubiosis, macrophages generally produce TGF-β, IL-6, and TNF; and T cells produce the pro-inflammatory Th17 cells by the differentiation of CD4 T cells; and thus, cause an adaptive immune response. The Th17 cell is abundant in the mucosal inflammation, which leads to CRC development [155]. The commensal bacteria, *Clostridia* species, can promote the overproduction of Th17 cells, leads to increased IL-17 generation in the epithelial cells. It is well established that Th17 acted as a driving force for the initiation of CRC Min-mouse models exposing the animals to enterotoxigenic *Bacteroides fragilis* [156] (Figure 4). These findings suggest that the inflammatory process plays a pivotal role among the gut microbiota and CRC. The pathogenic bacteria stimulate cancer formation through diverse mechanisms, including (a) dysbiosis and inflammation induced by a microorganism-associated molecular pattern (MAMP) triggering toll-like receptor (TLR) and additional pattern recognition receptors (PRR); (b) detrimental effects are intervened by bacterial toxins such as colibactin and CDT, and (c) acetaldehydes and nitrosamines by activating toxins through metabolic activities [157].

Nevertheless, the stimulus of the innate immunity achieved by toll-like receptor and agonists of the NOD-like receptor has been established as possible innate immunity and increase of anti-tumor activity [158]. Taking this into account, these explanations recommend an ambiguous role of the gut microbiota in carcinogenesis that may be reliant on the grade and mechanism. Microbial diversity is considerably poorer in tumor tissues matched with noncancerous tissues, proposing that a more appropriate microhabitat occurs in the vicinity to gut tissue. In CRC patients, the higher abundances of Erysipelotrichaceae, Prevotellaceae, Coriobacteriaceae, Lactobacillales, *Fusobacterium, Porphyromonas, Peptostreptococcus, Mogibacterium, Escherichia- Shigella,* *Prevotella* and lower loads of *Bifidobacterium, Faecalibacterium,* *Blautia, Staphylococcus*, and *Bacillus* were found [156,157,158]. These observations recommend certain bacteria may well compete in the converted niche and disclose novel steps in which the microbiota influences CRC development.

### Gut Microbiome Regulates Wnt/β-catenin Signaling Pathways

Adult CSC has the properties of self-renewal and targeting for cancer-originating mutation [159]. Elevated mutations in colon CSC ensuing the changes in variation/plasticity and site of the stem cell/propagation are the most represented primary sign for colon tumorigenesis [160]. The initiation of colon tumorigenesis is frequently determined by mutations in the Wnt signaling. Wnt is generally a secreted signaling protein. Conversely, the loss of function of adenomatous polyposis coli or gain of function of *β*-catenin causes the balance of unrestricted *β*-catenin that provides abnormal Wnt signaling leads to tumorigenesis [161]. Notably, mutation triggering of the Wnt pathway in G-protein-coupled receptor (Lgr5^+^) cells contributes to intestinal tumors with high competence compared to other colonic cell tumors [162]. According to the CSC hypothesis, the populace of colon cells can propagate tumor generation, measured as multipotent resulting in the cell of cancer [162,163]. Current data shows that multiple CSC hierarchies occur in the colon, facilitate cell fate in the account for various extrinsic factors including, diet, inflammation, and body anxiety [164]. Additionally, a function of diet in the maintenance of colon CSC has also described [165].

**Figure 5:**
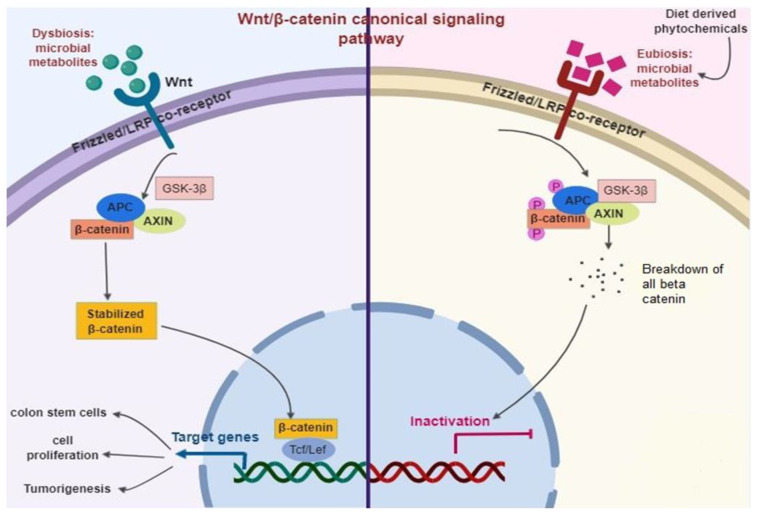
Diet-derived phytochemicals stabilize the microbiome status (Eubiosis) that inhibits Wnt/*β*-catenin signaling pathways successively prevent intestinal infection and inflammation.

The Wnt signaling generally occurs in an upstream of the *β*-catenin pathway [166] (Figure 5). Briefly, Wnt ligands largely fix with the complex of the Frizzled/LRP co-receptor, which triggers the canonical pathway. Axin, a Wnt signaling inhibitor protein is employed to the cell membrane, resulting in the inactivation of the adenomatous polyposis coli complex succeeding in the equilibrium of *β*-catenin. When Wnt is triggered, *β*-catenin is instantly soothed, allowing transfer to the nucleus and fixes with T cell factor and eventually elicits the expression of target genes. Among them, Leucine-rich repeat-containing Lgr5^+^ genes participated in stem cell proliferation [167].

The adenomatous polyposis coli is normally a tumor-suppressor protein that is mutated in almost 80% of CRC. Thus, the stimulation of Wnt/*β*-catenin is a primary biomarker of colitis-related CRC [168]. Diet-derived phytochemicals balance the microbiome status (Eubiosis), which inhibits Wnt/*β*-catenin signaling pathways and successively prevent intestinal infection and inflammation [96,169].

## 7. The Triangular Relationship between Phytochemicals, Gut Microbiome, and CSCs

**Figure 6:**
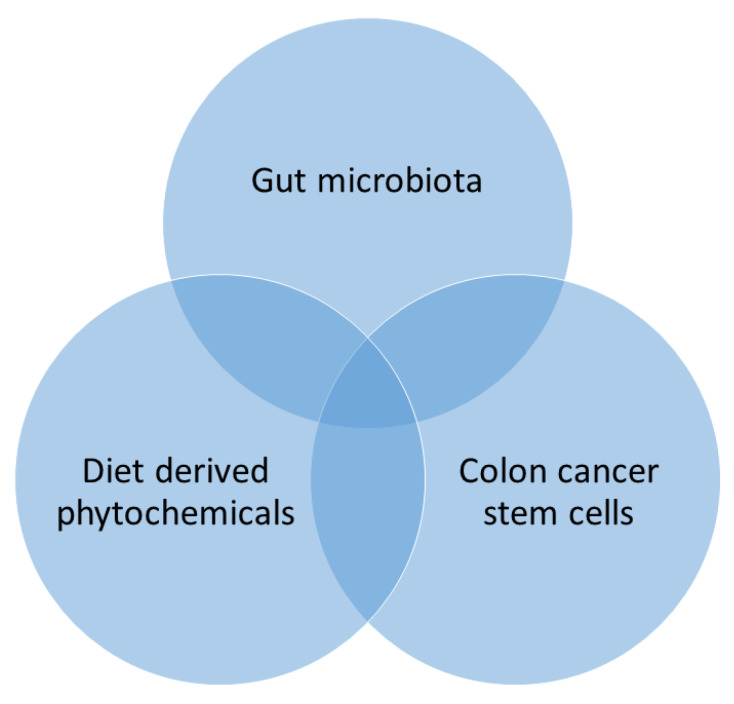
Triangular relationship between phytochemicals, gut microbiome, and cancer stem cells (CSCs).

Gut microbiota is chiefly affected by the dietary phytochemicals that can disturb its physiological relations in the host [170]. Through their alimentary canal route, phytochemicals are digested by colonic bacteria and produce several by-products [171]. These phytochemicals are rich in various active principles comprising polyphenols and flavonoids that upsurge the *Firmicutes, Bacteroidetes, Actinobacteria,* and *Proteobacteria* [30,171], which alters the pH of the colon environment and maintains the balance of the colonic microbiome [172]. Therefore, the effect of colonic bacteria on the dietary phytochemicals targeting dietary intervention which may contribute to host well-being [171]. The phytochemicals facilitate colonic bacteria, which may influence as adjuvants to treat cancer, obesity, diabetes, and chronic inflammatory diseases and prove as potentially prophylactics and candidates for the treatment of these diseases [30,171]. Furthermore, diet-derived phytochemical modulates colonic microbiota that targeting CSCs recognized as capable of decreasing the burden of CRC by triangle relationship (Figure 6).

The accumulating data put forward to the etiology of CRC, which is linked through the actions of colonic bacteria not only because of the pro-carcinogenic actions of particular pathogens but also other bacterial communities, especially their metabolome [173]. The multipotent colon CSCs undergo self-renewal during the asymmetric cell division and produce a populace of transit magnifying cells in CRC [174]. These cells undergo migration, proliferation, and differentiation to produce mature tumors and cancer progenitors. Uncontrolled proliferation or cell division of CSCs can repopulate [175].

Several phytochemicals including cinnamic acid, curcumin, EGCG, lycopene, quercetin, resveratrol, silibinin have been described to interfere with various regulatory pathways in the preservation of CSCs or to modify the CSC phenotype [12]. Notch, Hedgehog, and Wnt/*β*-catenin signaling pathways are the central signaling pathways, and they are involving in the self-renewal and differentiation of CSCs [102]. Thus, synergistic activities are anticipated when the CSC-directing phytochemicals and modulating colonic bacteria. By considering the above facts, CSCs influence a significant function in the tumor formation, targeting various signaling pathways and involves in the cancer development that may gain much interest in the field of cancer prevention via phytochemicals modulated colonic microbiota [176]. It is clearly understood that diet-derived phytochemicals undergo various alteration in the colonic bacteria and vice versa, various phytochemicals could regulate the colonic CSCs has also found to modify the gut microbiota population through triangular rapport, which may benefit to the host in combating the CRC.

## 8. Conclusions

CRC remains a significant threat to human society. Several investigations have elucidated the actions of several phytochemicals on the colon carcinogenesis via regulating several pathways, new insights into the relationships among the phytochemicals and colonic bacteria seem interesting and promising. Phytochemicals are a concoction of various bioactive compounds directing various cell signaling pathways that altered gut microbiota composition. This may support to destroying malignant cells with minor risks of emerging drug resistance. Dietary phytochemicals, or bioactive compounds and their analogs offer the advance of better-quality drugs that may ultimately provide the resolution to eradicate CSCs. These bioactive compounds would reinforce gut microbiota and combat against dreaded CRC. Dietary phytochemical-induced gut microbiota continues to be an encouraging and dynamic research niche in the upcoming days with evident anti-tumorigenesis effects and goals of abolishing the CSCs; propose novel opportunities for CRC prevention and treatment.

## Figures and Tables

**Table 1:** Effects of phytochemicals on gut microbiota and their anti-inflammatory effects.

Phytochemicals	Compounds	Model	Effect on Gut Microbiota	Anti-Inflammatory Effect	References
Anthocyanins	Anthocyanins	C57BL/6 J mice	Feces of gut microbiota-deficient mice showed an increase in anthocyanins and a decrease in their phenolic acid metabolites, while a corresponding increase was observed in jejunum tissue	Decreased the inflammatory status of mice	[49]
Anthocyanins	Anthocyanins	C57BL/6 J mice	Treatment modified the gut microbiota composition	Effectively reduced the expression levels of IL-6 and TNFα genes, markedly increased SOD and GPx activity	[50]
Catechins	Epigallocatechin-3-gallate	C57BL/6 J mice	The Firmicutes/Bacteroidetes ratio significantly lowered in HFD + EGCG, but higher in control diet + EGCG	Potential use for prevention, or therapy, for oxidative stress-induced health risks	[51]
Catechins	Epigallocatechin-3-gallate	C57BL/6 J mice	Maintained the microbial ecology balance and prevented dysbiosis	Suppressed the activation of NF-κB and decrease expression of iNOS	[52]
Catechins	Epigallocatechin-3-gallate	Wistar rats	Affected the growth of certain species of gut microbiota	Suppressed the activation of NF-κB	[53]
Catechins	Quercetin	C57BL/6 J mice	Increased Firmicutes/Bacteroidetes ratio and gram-negative bacteria and increased Helicobacter. Regulated gut microbiota balance	Reverted dysbiosis-mediated TLR-4, NF-κB signaling pathway activation, and related endotoxemia, with subsequent inhibition of inflammasome response and reticulum stress pathway activation	[54]
Catechins	Quercetin	Wistar rats	Attenuated Firmicutes/Bacteroidetes ratio, inhibiting the growth of bacterial species associated with diet-induced obesity (Erysipelotrichaceae, Bacillus, *Eubacterium cylindroides*). Quercetin was effective in lessening high-fat sucrose diet-induced gut microbiota dysbiosis	Suppressed the activation of NF-κB	[55]
Catechins	Quercetin	Fischer 344 rats	Exerted prebiotic properties by decreased pH, increased butyrate production, and altered gut microbiota	Suppressed the activation of NF-κB	[56]
Catechins	Kaempferol	3 T3-L1 adipocytes	Treatment modified the gut microbiota composition	Reduced LPS pro-inflammatory action, promoted anti-inflammatory and antioxidant effects	[57]
Flavonones	Baicalein	C57BL/6 J mice	Firmicutes/Bacteroidetes ratio significantly lowered and regulated dysbiosis	Suppressed the activation of NF-κB and decreased the expression of iNOS and TGF-β	[58]
Organosulfur compounds	Garlic essential oil and Diallyl disulfide	C57BL/6 mice	Treatment modified the gut microbiota composition	Significantly decreased the release of pro-inflammatory cytokines in the liver, accompanied by elevated antioxidant capacity via inhibition of cytochrome P450 2E1 expression	[59]
Phenolic acid	Curcumin	Mice	A direct effect of bioactive metabolites reaching the adipose tissue rather than from changes in gut microbiota composition	Nutritional doses of *Curcuma longa* decreased proinflammatory cytokine expression in subcutaneous adipose tissue	[60]
Phenolic acid	Curcumin	LDLR−/− mice	Improved intestinal barrier function and prevented the development of metabolic diseases	Significantly attenuated the Western diet-induced increase in plasma LPS levels	[61]
Phenolic acid	Curcumin	Human IEC lines Caco-2 and HT-29	Modulated chronic inflammatory diseases by reducing intestinal barrier dysfunction despite poor bioavailability	Significantly attenuated LPS-induced secretion of master cytokine IL-1β from IEC and macrophages. Reduced IL-1β-induced activation of p38 MAPK in IEC and subsequent increase in the expression of myosin light-chain kinase	[62]
Polyphenols	Polyphenols	C57BL/6 J ApcMin mice	Bacterial diversity was higher in the bilberry group than in the other groups	Attenuation of inflammation in cloudberry-fed mice	[63]
Stilbenes	Resveratrol	Kunming mice	HF microbiomes were different from those in CT and HF-RES mice. After treatment, Lactobacillus and Bifidobacterium were significantly increased, whereas *Enterococcus faecalis* was significantly decreased, resulting in a higher abundance of Bacteroidetes and a lower abundance of Firmicutes	Decreased the inflammatory status of mice	[64]
Stilbenes	Resveratrol	Glp1r−/− mice	Treatment modified the gut microbiota composition	Decreased the inflammatory status of mice	[65]
Stilbenes	Resveratrol	Wistar rats	Trans-resveratrol supplementation alone or in combination with quercetin scarcely modified the gut microbiota profile but acted at the intestinal level, altering mRNA expression of tight-junction proteins and inflammation-associated genes	Altered mRNA expression of tight-junction proteins and inflammation-associated genes	[55]
Stilbenes	Resveratrol	Adipocytes	Treatment modified the gut microbiota composition	Resveratrol opposed the effect induced by LPS, functioning as an ameliorating factor in disease state	[66]
Stilbenes	Resveratrol	Human	Steroid metabolism of the affected gut microbiota was studied	-	[67]
Stilbenes	Piceatannol	C57BL/6 mice	Altered the composition of the gut microbiota by increasing Firmicutes and Lactobacillus and decreasing Bacteroidetes	Decreased the inflammatory status of mice	[68]
Stilbenes	Piceatannol	Zucker obese rats	It did not modify the profusion of the most abundant phyla in gut microbiota, though slight changes were observed in the abundance of several Lactobacillus, Clostridium, and Bacteroides species belonging to Firmicutes and Bacteroidetes	Showed a tendency to reduce plasma LPS by 30%	[69]

Abbreviation: Caco-2—human epithelial colorectal adenocarcinoma cells; CT—control diet; EGCG—Epigallocatechin-3-gallate; GPx—glutathione peroxidase; HF-RES—high-fat diet supplemented with resveratrol; HFD—high-fat diet; IEC—intestinal epithelial cells; IL 6—interleukin 6; iNOS—inducible nitric oxide synthase; LPS—lipopolysaccharides; MAPK—mitogen-activated protein kinase; mRNA—messenger ribonucleic acids; NF-κB—nuclear factor kappa B; SOD—superoxide dismutase; TGF β—transforming growth factor-beta; TLR-4—toll-like receptor 4; TNFα—tumor necrosis factor-alpha; P450 2E1—cytochrome P450 2E1.

**Table 2:** Tumorigenic effects of colon cancer stem cells (CSCs).

Disease or Model	Cell Surface Markers	Findings	Mechanisms	References
AOM in *Il10*^−/−^ gnotobiotic mice	CD133, CD44, ALDH1 CD166, EpCAM, CD24, CD29	Tumor detection in the mice	TNF-α and NO-mediated dysbiosis, barrier failure, chronic inflammation, bacterial genotoxicity	[94]
AOM plus DSS -treated mice treated with an antibiotic cocktail		Tumor detection in the antibiotic-treated mice		[95]
AOM-induced		Tumor detection in the rats		[96]
AOM-induced		Tumor detection in the rats		[97]
*Apc*^Min/+^*Cdx2*–Cre mice treated with an antibiotic cocktail		Tumor detection in the antibiotic-treated mice		[95]
*Apc*^Min/+^ mice		Tumor detection in the mice		[98]
DMH-induced		Tumor detection in the rats		[96]
MAM-GlcUA- induced		Tumor detection in the rats		[96]
*Nod1*^−/−^ mice treated with an antibiotic cocktail		Tumor detection in the antibiotic-treated mice		[99]
Spontaneous carcinogenesis		Tumor detection in the rats		[96]
Wild-type microbiota transplanted into *Nod2*^−/−^mice		Tumor detection in the after transplant		[100]

Abbreviation: AOM-azoxymethane; Apc^Min^—adenomatous polyposis coli/multiple intestinal neoplasia; CD—a cluster of differentiation; Cdx2—human caudal type homeobox 2; DMH- 1,2-Dimethylhydrazine; DSS—dextran sodium sulfate; EpCAM—epithelial cell adhesion molecule; EphB—ephrin B; MAM-GlcUA—methyl azoxy methanol-beta-D-lucosiduronic acid; NO—nitric oxide; Nod—nucleotide-binding oligomerization domain-containing protein.

**Table 3:** List of phytochemicals and their anti-tumorigenic effect on colon CSC.

Dietary Phytochemical	Sources	Molecular Mechanistic Action	References
(+)-catechin, chlorogenic acid, ellagic acid, and gallic acid	Walnut phenolic extract (WPE)	WPE down-regulated the CSC markers such as CD133, DLK1, CD44, and Notch1. WPE downregulated the β-catenin/p-GSK3β signaling pathway. The CSC’s self-renewing capacity was suppressed by WPE. Overall, WPE regulated the characteristics of colon CSCs.	[117]
Cinnamic acid	Fruits, vegetables, and whole grains	Cinnamic acid reduced the CSC markers associated with HT-29 colon cancer cells.	[119]
Curcumin	Turmeric	Curcumin decreased the ALDH1 activity, decreases CD44+, CD133+, CD166+ cell numbers, and induces apoptosis. Induces G2/M phase arrest, and downregulates the expression of *β*-catenin.	[113,114]
EGCG	Apple skin, green and black tea, onions, carob, plums, hazelnuts, and pecans.	EGCG suppressed glycoprotein; reduced the expression Wnt signaling, cell cycle, Hedgehog, Akt/mTOR, NF-κB, and VEGF pathways; Induced apoptosis.	[126]
Lycopene	Olive, tomatoes, watermelon, pink grapefruit, pink guava, papaya, seabuckthorn, wolfberry, and rosehip	Downregulated Akt/mTOR, and VEGF, Epigenetic alterations	[127]
p-Coumaric Acid and tricin	Sasa quelpaertensis extract (SQE)	Induced CSC differentiation and inhibited Wnt signaling. Suppressed the expression of CSC markers, hypoxia-inducible factor-1α (HIF-1α) signaling, and Wnt/*β*-catenin signaling.	[115]
Quercetin	Leafy vegetables, broccoli, red onions, peppers, apples, grapes, black and green tea, red wine	Induced apoptosis, and downregulated Wnt, Hedgehog, NF-κB, PI3K/Akt, MRP1, 4, and 5	[128]
Resveratrol	Peanuts, pistachios, grapes, wine, blueberries, cranberries, cocoa, and dark chocolate	Resveratrol acted on colon CSCs via inhibition of Wnt signaling	[116]
Silibinin	Milk thistle seeds	Silibinin acted via pro-tumorigenic signaling blocking and IL-4/-6 signal blocking; Suppressed the activation of the PP2Ac/AKT Ser473/mTOR pathway; Inhibited tumor formation rate, tumor growth, and colon CSLCs self-renewal.	[92,118]
Sulforaphane	Broccoli Sprouts, Cauliflower, Cabbage, Brussels Sprout, Bok Choy, Collards	Reduced the expression of NF-κB, Akt/mTOR, ALDH1, Wnt signaling, Induced apoptosis, downregulated epithelial-mesenchymal transition	[129]

Abbreviation: Akt—serine/threonine-specific protein kinase; Aldh1—Aldehyde Dehydrogenase 1; CD—a cluster of differentiation; CSCs—colon cancer stem cells; DLK1—Delta Like Non-Canonical Notch Ligand 1; EGCG—epigallocatechin gallate; GSK3β—glycogen synthase kinase 3 beta; HT-29—human colorectal adenocarcinoma cells; IL—interleukin; MRP- Multidrug resistance-associated protein; mTOR—mammalian target of rapamycin; NF-κB—nuclear factor kappa-B; PP2Ac- Protein phosphatase 2A homologs, catalytic domain; VEGF—vascular endothelial growth factor.

## Abbreviations

Akt	serine/threonine-specific protein kinase
Aldh1	aldehyde Dehydrogenase 1
AOM	azoxymethane
AP1	activator protein 1
APC	adenomatous polyposis coli
Apc^Min^	adenomatous polyposis coli/ multiple intestinal neoplasia
ASK1	apoptosis signal-regulating kinase 1
b.w.	body weight
Bax	bcl-2-like protein
Bcl 2	B-cell lymphoma 2
Bid	BH3 Interacting domain death agonist
Caco-2	human epithelial colorectal adenocarcinoma cells
CD	cluster of differentiation
Cdx2	human caudal type homeobox 2
CIP1/waf1	cyclin-dependent kinase inhibitor 1
c-MYC	myc protein
CRC	colorectal cancer
CSCs	colon cancer stem cells
Cyt C	cytochrome C
DLK1	delta like non-canonical notch ligand 1
DMH	1,2-Dimethylhydrazine
DNA	deoxyribonucleic acid
DNMT	DNA methyltransferase
DSS	dextran sodium sulfate
EGCG	Epigallocatechin-3-gallate
EGFR	epidermal growth factor receptor
EpCAM	epithelial cell adhesion molecule
EphB	ephrin B
ESA	epithelial surface antigen
FAK	Focal adhesion kinase
GPx	glutathione peroxidase
GSK3	glycogen synthase kinase-3
HAT	histone acetyltransferases
HDAC	histone deacetylase
HFD	high-fat diet
Hh	hedgehog
HT-29	human colorectal adenocarcinoma cells
IBD	chronic inflammatory bowel disease
IEC lines	intestinal epithelial cell lines
IL	interleukin
iNOS	inducible nitric oxide synthase
JNK	c-Jun N-terminal kinases
Kip1	kinesin-like protein1
LDLR	low-density lipoprotein receptor
LGR5	leucine-rich repeat-containing G protein-coupled receptor 5
LPS	lipopolysaccharides
MAM-GlcUA	methyl azoxy methanol-beta-D-glucosiduronic acid
MAPK	mitogen-activated protein kinase
MKK4	mitogen-activated protein kinase kinase 4
mRNA	messenger ribonucleic acid
MRP	Multidrug resistance-associated protein
mTOR	mammalian target of rapamycin
NF-κB	nuclear factor kappa-B
NO	nitric oxide
Nod	nucleotide-binding oligomerization domain-containing protein
PDGF	platelet-derived growth factor
PDGFRα	platelet-derived growth factor receptor A
PI3K	Phosphoinositide 3-kinases
PP2Ac	Protein phosphatase 2A homologs, catalytic domain
siRNA	Small interfering RNA
SOD	superoxide dismutase
SrC	protooncogene c
STAT3	signal transducer and activator of transcription 3
Tcf4	T-cell factor 4
TGFα	Transforming Growth Factor-alpha
TLR-4	Toll-like receptor 4
TNF-α	tumor necrosis factor-alpha
VEGF	vascular endothelial growth factor
VEGFR	vascular endothelial growth factor receptor
